# Correlation between intervertebral disc degeneration and bone mineral density difference: a retrospective study of postmenopausal women using an eight-level MRI-based disc degeneration grading system

**DOI:** 10.1186/s12891-022-05793-w

**Published:** 2022-09-03

**Authors:** Yihui Zhang, Patiman, Beibei Liu, Rui Zhang, Xiaofei Ma, Hui Guo

**Affiliations:** 1 Department of Radiology, Xinjiang Medical University Affiliated Sixth Hospital, Urumqi, 830002 Republic of China; 2 Medical Imaging Center, Xinjiang Medical University Affiliated First Hospital, Urumqi, 830054 Republic of China

**Keywords:** Postmenopausal women, BMD difference, Intervertebral disc degeneration, Modic change, Osteoporosis, MRI

## Abstract

**Purpose:**

To explore the correlation between intervertebral disc degeneration (IDD) and bone mineral density (BMD) difference between adjacent vertebrae.

**Methods:**

A retrospective analysis of 114 postmenopausal women who were treated in our hospital from January 2021 to December 2021. The degree of lumbar(L)1–5 IDD was scored according to an 8-grade scoring system. The lumbar vertebrae BMD was detected, and the BMD difference was calculated. The subjects were grouped according to age and whether the disc was severe IDD. Data were collected for statistical analysis.

**Results:**

The prevalence of osteoporosis in the 51–60-year-old group was lower than that in the other groups, while the prevalence of modic changes in the 71–80-year-old group was higher than that in the 51–70-year-old group (*P* < 0.05). At the L1/2 level, the prevalence of severe IDD in the 81-90y group was higher than that in the 51-70y group (*P* < 0.05). At the L2/3 level, the prevalence of severe IDD in the 71-90y group was higher than that in the 51-60y group, and the prevalence of severe IDD in the 71-80y group was higher than that in the 61-70y group (*P* < 0.05). The L2/3 disc score was positively correlated with the L3-L2 BMD difference (*P* < 0.05). At the level of L1-2, the BMD difference in the non-severe IDD group was smaller than that in the severe IDD group (*P* < 0.05).

**Conclusion:**

For postmenopausal women, an increase in BMD difference is correlated with IDD. Osteoporosis is more common in people over 60 years old, and the possibility of modic change in 71-80y is higher than in other age groups. The incidence of severe IDD also increases with aging, especially for the L1/2 and L2/3 discs.

## Background

Intervertebral disc degeneration(IDD) is a very common disease among the elderly, and it is also one of the important causes of low back pain in adults. IDD can lead to disc herniation, spinal stenosis, and degenerative spondylolisthesis, which can cause low back pain. In the most severe cases, IDD can lead to complete incapacity and disability in patients. Spinal degenerative diseases often accompany osteoporosis with aging, especially in postmenopausal women. Postmenopausal women also suffer from more severe IDD than men of the same age due to the rapid decline in bone mineral density(BMD) caused by the dramatic drop in estrogen levels in their bodies [1].

There are differences in BMD between adjacent vertebral bodies. One study showed that lumbar(L) 1 to L4 and bone mineral content increased gradually [2]. The study by Hayashi T et al. showed that the trabecular BMD of L3 was the lowest relative to other vertebral levels. In all age groups, BMD tended to increase gradually from L3 to Thoracic(T) 1 [3]. Another study reported that BMD in both men and women tended to decrease gradually from T1 to L3, followed by a gradual increase from L4 to L5 [4]. There are few studies on the BMD difference between adjacent lumbar vertebrae. In recent years, there have been some studies on the T-score/Z-score difference between adjacent lumbar vertebrae. Lajlev SE et al. randomly found that a T-score difference ≥ 1.5 SD between adjacent vertebrae was associated with a small increase in the risk of compression fractures when using Dual-energy X-ray absorptiometry (DXA) [5]. Another study involving 5–18 years showed that differences in Z-scores for lumbar spine BMD were not associated with lumbar fractures in children and adolescents. In the absence of fractures, differences in Z-scores may represent variability in vertebral development in children whose bones are still growing [6].

There are many factors that contribute to IDD. One study showed that aging, high BMI, high low-density lipoprotein cholesterol (LDLc), occupational weightlifting and physical activity were associated with lumbar intervertebral disc degeneration(LIDD) in older adults [7]. BMD is also one of the factors associated with IDD. Yue Wang et al. used microCT to remove osteophytes and cartilage endplates and found that increased BMD was significantly associated with more severe LIDD [8]. As we all know, the intervertebral disc is located between the adjacent upper and lower vertebral bodies. The intervertebral disc which is flexible and elastic plays a role in balancing and buffering shocks to the adjacent vertebral bodies.

Is the BMD difference between adjacent vertebrae related to the degeneration of the corresponding intervertebral disc? Can BMD difference be used as a predictor of spinal disc degeneration? Our study investigated whether there was a correlation between IDD and the BMD difference between adjacent vertebrae in postmenopausal women.

## Material and methods

### Subjects

A total of 132 postmenopausal women who visited the Sixth Affiliated Hospital of Xinjiang Medical University from January 2021 to December 2021 were enrolled. Inclusion criteria: women over 50 years old, postmenopausal, with clinical syndromes of lumbar disc degeneration such as chronic low back pain, completed Magnetic resonance imaging(MRI) and BMD examination (the interval between two examinations should not exceed 3 months). Based on imaging and electronic medical records, we excluded 2 cases of severe scoliosis, 4 cases of lumbar hemangioma, 1 case of vertebral metastases, 3 cases of lumbar internal fixation, 2 cases of lumbar bone cement surgery, 2 cases of recent lumbar fractures, 2 cases of recent lumbar fractures, 3 cases who had taken calcitriol or alendronate sodium tablets for 3 months and 1 case who had hypothyroidism. Finally, 114 postmenopausal women aged 68.0 (54–87) years were included in the study.

### Measurement of bone mineral density

Lumbar spine BMD was detected by dual-energy X-ray absorptiometry (GE, Lunar Prodigy). Before measuring BMD, we first performed quality assurance (QA) testing and calibrated the dual-energy X-ray absorptometer with the module to evaluate the stability of the system. Before measuring BMD, all subjects were placed in a standard body position. After BMD scanning, the software system automatically obtained the BMD and T values of L1-L4. When the position of the vertebral line of the lumbar spine analysis chart was not suitable, we manually adjusted it. Each subject was defined as normal bone mass (T-score > -1), osteopenia (-2.5 < T-score ≤ -1) or osteoporosis (T-score ≤ -2.5), according to lumbar spine T-score and World Health Organization criteria. We calculated the BMD difference at L2-L1, L3-L2, and L4-L3 levels. It was calculated by subtracting the BMD of the upper lumbar vertebra from the BMD of the lower lumbar vertebra.

### MRI scan and disc degeneration score

The lumbar spine of the subjects was scanned with a 1.5 T MRI scanner(Siemens, Germany). It has been reported that inter-rater and intra-rater reliability for grading intervertebral discs according to the modified Pfirrmann system is very high (Intrareader-weighted kappa range:0.79–0.91; Interreader-weighted kappa range:0.65–0.67) [9]. Before using the modified Pfirrmann system, face-to-face training was conducted by 2 MRI radiologists. Then, the L1/2 to L4/5 discs (456 discs in total) were assessed on T2-weighted sagittal lumbar MRI using the modified Pfirrmann grading system.

This grading system for disc degeneration is based on disc MR signal intensity, disc structure, the distinction between nucleus and annulus, and disc height. With this grading system, grade 1 corresponds to no IDD; grade 2 corresponds to mild IDD; grade 4/5 corresponds to moderate IDD; grade ≥ 6 indicates an existence of disc space narrowing, while grade 8 corresponds to end-stage degeneration [9]. Grades 1–5 were defined as "mild-to-moderate IDD", whereas grades 6–8 were defined as "severe IDD". There were individual cases showing lumbar disc hyperintensity accompanied by a decrease in disc height. At this time, we scored IDD based on the height of the disc.

In addition, we observed the presence of spondylolisthesis, modic changes and Schmorl’s nodes by MRI sagittal images. Spondylolisthesis was defined as an anterior or posterior sliding of the superior vertebral body relative to the inferior vertebral body. Modic changes were defined as abnormal signals (type I: Low signal on T1WI and high signal on T2WI; type II: High signal on T1WI and iso- to high signal on T2WI; type III: Low signal both on T1WI and T2WI) in the vertebral endplate margins. Schmorl’s node was defined as a bony defect at the edge of the vertebral body endplate. The subchondral disc of the vertebral body was interrupted and disappeared, and the signal of the lesion was close to the nucleus pulposus of the intervertebral disc.

### Statistical analysis

Statistical analysis was performed using SPSS 19.0 statistical analysis software. The Kolmogorov–Smirnov test was used to test whether the data conformed to a normal distribution. The homogeneity of variance for the measurement data was assessed using the Levene test. Measurement data and grade data were expressed as mean(standard deviation). Enumeration data were expressed as numbers (percentages). Enumeration data were compared using a chi-square test or fisher's exact test. A comparison of the measurement data was carried out by an independent sample t-test. The correlation analysis between the intervertebral disc score and the BMD difference between adjacent vertebrae was performed using spearman correlation analysis. When the P value was less than 0.05, the difference was statistically significant.

## Results

### Baseline data

**Fig. 1:**
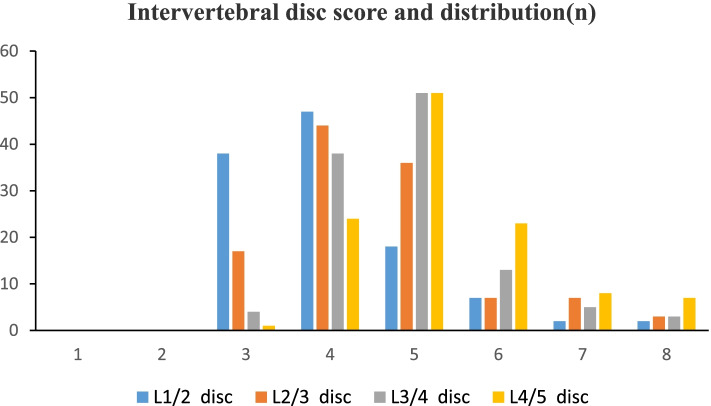
Column graph showing differences in disc number for different disc scores. The ordinate represented the number of intervertebral discs, and the abscissa represented the modified Pfirrmann system score. All study subjects had disc scores between 3 and 8

All subjects had 456 intervertebral discs with scores ranging from 3 to 8. There were 60 (13.2%) discs of grade 3. There were 153 (33.6%) discs of grade 4. There were 156 (34.2%) discs of grade 5. There were 50 (11.0%) discs of grade 6. There were 22 (4.8%) discs of grade 7. There were 15 (3.3%) discs of grade 8, as shown in Fig. 1.

**Fig. 2:**
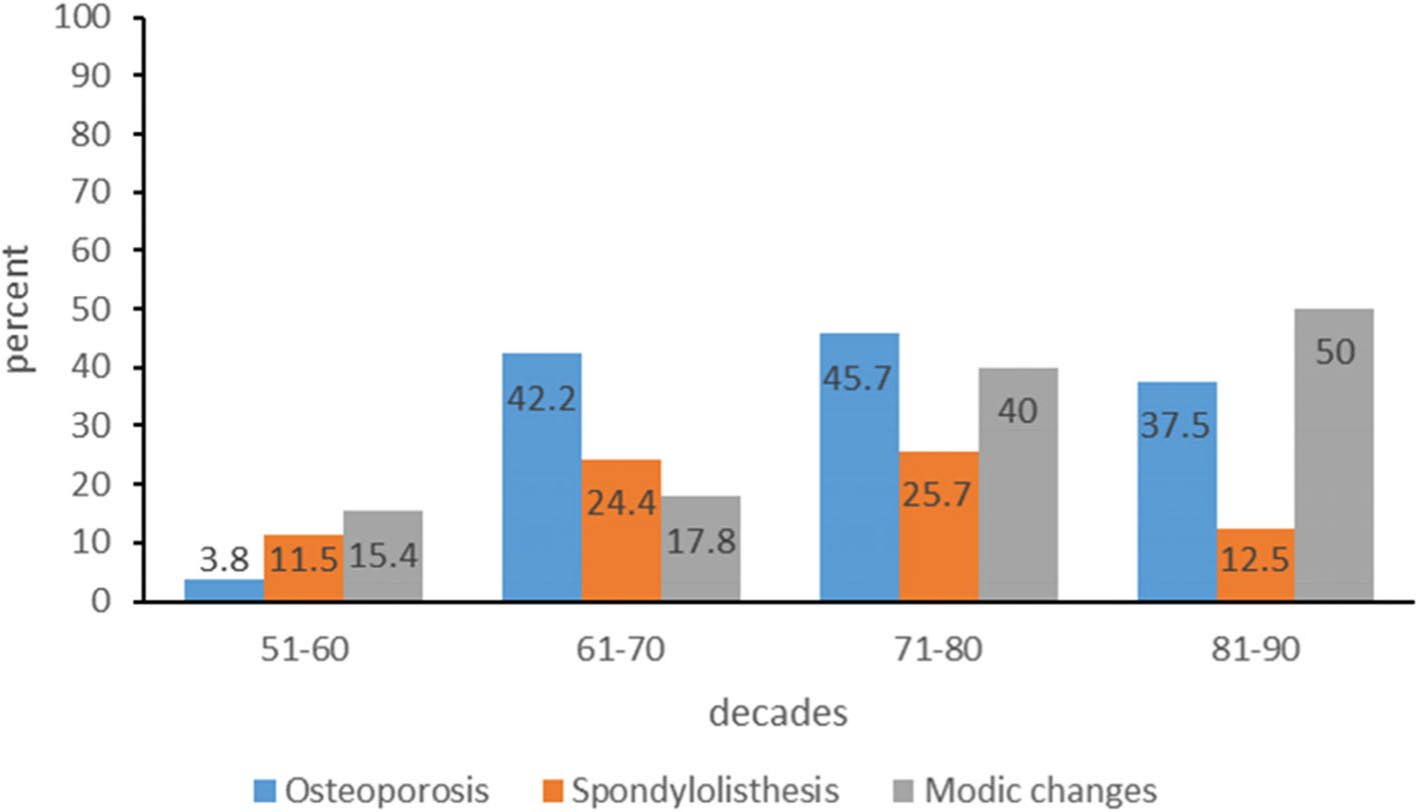
Column chart showing the differences in the prevalence of osteoporosis, spondylolisthesis and modic changes by age group.The vertical axis represented prevalence, expressed as percentage. The horizontal axis represented each age group

We divided the subjects into 4 groups by age, and the baseline data after grouping are shown in Table 1. We compared the prevalence of osteoporosis, spondylitis, spondylolisthesis and severe IDD(grade ≥ 6) in each age group. The prevalence of osteoporosis in the 51-60y group was lower than that in other groups (Chi-square value (51–60 vs. 61–70) = 11.994, Chi-square value (51–60 vs. 71–80) = 13.009, Chi-square value (51–60 vs. 81–90) = 6.675, *P* < 0.05), and there was no statistical difference in the prevalence of osteoporosis among other groups (*P* > 0.05). There was no significant difference in the prevalence of spondylolisthesis among different groups (*P* > 0.05). The prevalence of modic changes in the 71-80y group was higher than that in the 51-70y group (Chi-square value (51–60 vs. 71–80) = 4.346, Chi-square value (61–70 vs. 71–80) = 4.876, *P* < 0.05), and the prevalence of modic changes in other age groups was not statistically different (*P* > 0.05), as shown in Fig. 2. Only 1 case of Schmorl’s nodes was seen in the 61-70y group.

**Table 1:** Baseline data

	**Decades**			
	**51-60y**	**61-70y**	**71-80y**	**81-90y**
**n**	26	45	35	8
**Age(y)**	57.7(1.9)	65.4(2.5)	75.9(2.8)	82.5(2.1)
**Age of menopause(y)**	50.2(3.0)	49.0(5.1)	48.3(2.7)	48.8(3.6)
**Height(m)**	1.64(0.06)	1.61(0.06)	1.57(0.06)	1.53(0.05)
**Weight(kg)**	66.9(13.1)	64.3(8.4)	61.1(11.6)	58.1(7.6)
**Body mass index(kg/m**^**2**^**)**	24.6(3.5)	24.8(3.5)	24.9(4.4)	24.9(4.2)
**L1 BMD(g/cm**^**2**^**)**	1.027(0.142)	0.858(0.178)	0.858(0.208)	0.857(0.150)
**L2 BMD(g/cm**^**2**^**)**	1.066(0.122)	0.932(0.167)	0.903(0.219)	0.929(0.206)
**L3 BMD(g/cm**^**2**^**)**	1.146(0.152)	1.014(0.200)	0.959(0.215)	1.001(0.214)
**L4 BMD(g/cm**^**2**^**)**	1.147(0.182)	1.039(0.202)	1.006(0.239)	1.079(0.236)
**L2-L1 BMD difference(g/cm**^**2**^**)**	0.038(0.076)	0.074(0.087)	0.045(0.080)	0.072(0.082)
**L3-L2 BMD difference(g/cm**^**2**^**)**	0.080(0.087)	0.082(0.101)	0.056(0.077)	0.072(0.086)
**L4-L3 BMD difference(g/cm**^**2**^**)**	0.001(0.108)	0.026(0.094)	0.046(0.093)	0.078(0.087)
**T-score(SD)**	-0.8(1.0)	-2.0(1.3)	-2.1(1.6)	-2.2(1.0)

**Fig. 3:**
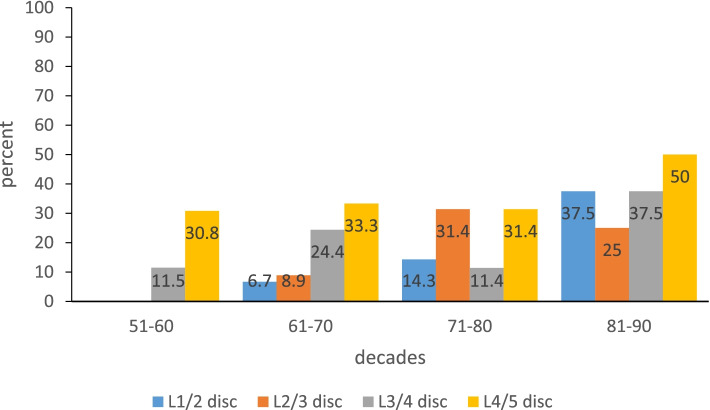
The bar graph showed the difference in the prevalence of severe IDD in different age groups. The vertical axis represented prevalence, expressed as percentage. The horizontal axis represented each age group

For L1/2 intervertebral disc, the prevalence of severe IDD in the 81-90y group was higher than that in the 51-70y groups (Chi-square value (51–60 vs. 81–90) = 10.694, Chi-square value (61–70 vs. 81–90) = 6.432, *P* < 0.05), and there was no significant difference in the prevalence of severe IDD among other age groups (*P* > 0.05). For L2/3 intervertebral disc, the prevalence of severe IDD in the 71-90y group was higher than that in the 51-60y group (Chi-square value (51–60 vs. 71–80) = 9.969, Chi-square value (51–60 vs. 81–90) = 6.906, *P* < 0.05), and the prevalence of severe IDD in the 71-80y group was higher than that in the 61-70y group (Chi-square value (61–70 vs. 71–80) = 6.565, *P* < 0.05). For L3/4 and L4/5 intervertebral discs, there was no statistical difference in the prevalence of severe IDD in each age group (*P* > 0.05), as shown in Fig. 3.

### Correlation between IDD and BMD difference

**Fig. 4:**
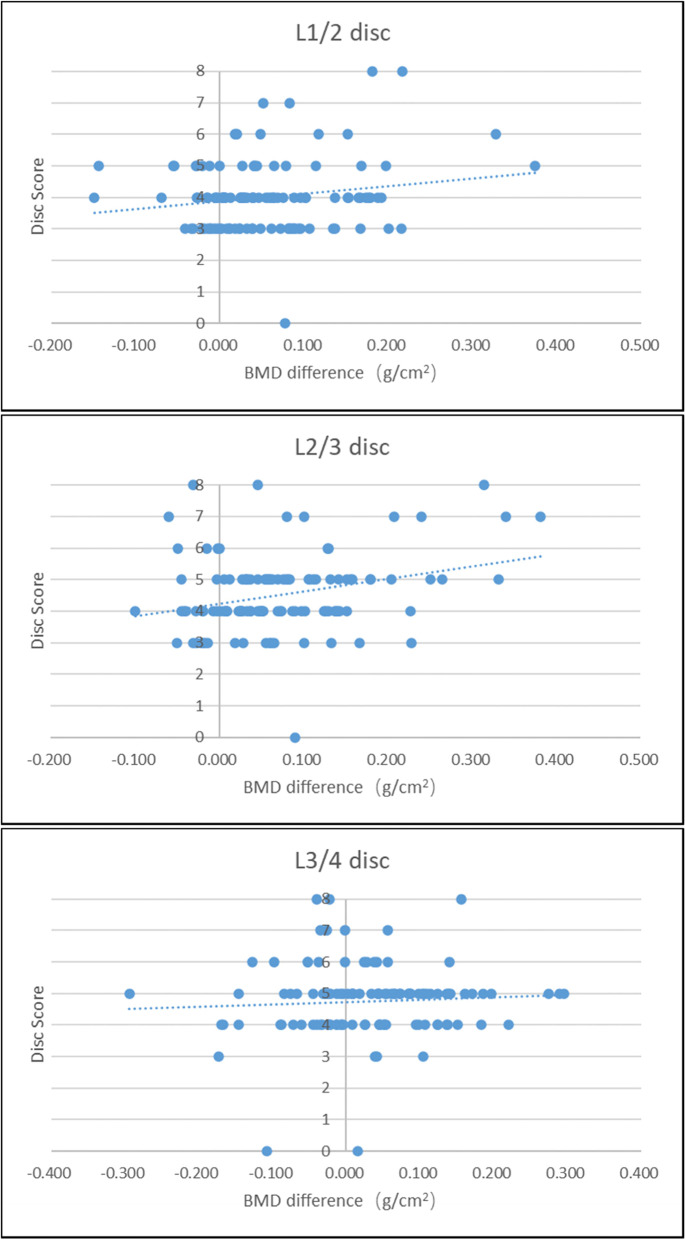
The three scatter plots showed correlation of the L1-4 intervertebral disc score with the corresponding BMD difference respectively

The score of the L2/3 intervertebral disc was positively correlated with the L3-L2 BMD difference (spearman coefficient = 0.261, *P* = 0.005). This indicates that the BMD of L3 was higher than that of L2, and the degeneration of L2/3 disc was severe. There was no correlation between the L1/2 intervertebral disc score and the L2-L1 BMD difference (spearman coefficient = 0.103, *P* = 0.274), and there was no correlation between the L3/4 intervertebral disc score and L4-L3 BMD difference (spearman coefficient = -0.015, *P* = 0.870), as shown in Fig. 4.

We divided the L1/2, L2/3 and L3/4 intervertebral discs into two groups: non-severe IDD (score < 6) and severe IDD(score ≥ 6). The results showed that at the level of L1-2, the BMD difference in the non-severe IDD group was smaller than that in the severe IDD group (t value =-2.606, *P* < 0.05), as shown in Table 2.

**Table 2:** Relationship between severe IDD and BMD difference

Disc	group	Number	BMD difference	t value	p value
L1/2	non-severe IDD	103	0.051 (0.079)	-2.606	0.01*
	severe IDD	11	0.117 (0.095)		
L2/3	non-severe IDD	97	0.066 (0.077)	-1.316	0.205
	severe IDD	17	0.111 (0.140)		
L3/4	non-severe IDD	93	0.037 (0.102)	1.669	0.098
	severe IDD	21	-0.002(0.072)		

## Discussion

IDD seriously affect people's health, and the cost of their treatment has caused a huge economic burden to the society. IDD and osteoporosis are very common in postmenopausal women. It is well known that postmenopausal women's BMD declines much faster than men of the same age due to declining estrogen levels. Lou et al. demonstrated through a large sample study that estrogen deficiency may be a risk factor for LIDD [10].

Our study found that the osteoporosis prevalence in the 51-60y group was lower than in other groups (*P* < 0.05). This suggests that the prevalence of osteoporosis in postmenopausal women under the age of 60 is lower than that in postmenopausal women over the age of 60. This study also found that the prevalence of modic changes in the 71-80y group was higher than that in the 51-70y group (*P* < 0.05). This indicates that modic changes are easily seen in the lumbar spine of postmenopausal women aged 71-80y. The study by Tarukado K et al. showed that the prevalence of modic changes increased with age, with the highest incidence in people in 60 s and a decrease in the incidence in people in 70 s and 80 s [11]. Modic changes are also closely related to IDD. The study by Ozcan-Eksi EE showed that modic change was closely associated with severe IDD at all corresponding lumbar levels except for the L1-2 [12]. Schmorl’s nodes were uncommon in the lumbar spine of postmenopausal women in this study. Only 1 case of a 65-year-old female had Schmorl’s nodes at the edge of the endplates of the L2-L3 vertebral bodies. Schmorl’s nodes are closely associated with severe IDD at the L1-L2 and L2-L3 levels [13]. Some studies have also suggested that Schmorl’s nodes are associated with the anterior degeneration of the annulus fibrosus in patients over 50 [14].

The Pfirrmann grading system has been widely accepted and used clinically as a standard for evaluating IDD [15]. At the beginning of this study, we used the Pfirrmann grading system to score IDD and often encountered ambiguity. Therefore, most disc scores were 3 or 4. The reason may be that all subjects in this study were postmenopausal women over the age of 54 years, and the Pfirrmann grading system couldn’t distinguish the subtle differences between degenerative discs. This study finally adopted the modified Pfirrman grading system, which was more suitable for evaluating IDD in the elderly [9]. In the modified Pfimmann grading system, grade 3 is divided into two grades (modified grades 3 and 4), and grade 4 is divided into three grades (modified grades 5, 6, and 7). The results of this study showed that at the L1/2 level, the prevalence of severe IDD in the 81-90y group was higher than that in the 51-70y group (*P* < 0.05). At the L2/3 level, the prevalence of severe IDD in the 71-90y group was higher than that in the 51-60y group (*P* < 0.05). The prevalence of severe IDD in the 71-80y group was higher than that in the 61-70y group with severe IDD (*P* < 0.05). This suggests that age is a risk factor for developing severe IDD, especially for L1/2 and L2/3 discs.

Our study found that the BMD of L3 was higher than that of L2, and the degeneration of L2/3 disc was severe. In addition, at the level of L1-2, the BMD difference in the severe IDD group was greater than that in the non-severe IDD group (*P* < 0.05). These results all indicate that an increase in the BMD difference is correlated to IDD. Fein et al. believed that there was a potential direct correspondence between the pressure in the area above or below the nucleus pulposus of the intervertebral disc and BMD of the vertebral body, and biomechanics indicated that changes in the pressure distribution within the intervertebral disc would affect the BMD distribution of the adjacent vertebral bodies [16]. A finite element study showed that transferring vertebral and intervertebral disc pressure loads involved more than just the axial transfer of forces along each sagittal column [17]. We speculate that when BMD loss of the upper vertebral body exceeds BMD loss of the lower vertebral body to a certain critical value, pressure load distribution of the corresponding intervertebral disc will change at L1-2 and L2-3 levels. This can cause microcirculation disturbance and insufficient blood supply in the intervertebral disc, and accelerate IDD. There was no significant correlation between BMD difference and L3-4 IDD. This may be related to the increase in the anteversion angle of the L4 vertebral body and the transmission direction change of the pressure load between the L3/4 intervertebral disc and the endplate of the L4 vertebral body. So far, there have been several studies on the correlation between BMD and IDD. Some studies suggest that they are positively correlated. The study by Zhou L et al. showed that lumbar spine BMD in postmenopausal women was positively correlated with LIDD, especially in the upper lumbar spine (L1, L2) [18]. Another study showed that higher lumbar spine BMD/Z scores were associated with more severe LIDD [19]. Some studies have come to the opposite conclusion. Homminga J et al. showed that LIDD could lead to a decrease in BMD in the center of trabecular bone. IDD and osteoporosis have a synergistic effect on vertebral fractures [20]. Pan et al. conducted a statistical analysis of 512 Chinese patients and found that BMD was not a risk factor for LIDD in Chinese. There is a significant correlation between lumbar facet joint osteoarthritis and BMD. As lumbar facet osteoarthritis affects the outcomes of lumbar spine BMD, this may confound the link between spine BMD and LIDD [21].

There are several theories describing the correlation between BMD and IDD. The intervertebral disc contains almost no blood vessels, and only a small amount of tiny capillaries grow into the outer annulus fibrosus. The main nutrient supply of the intervertebral disc still comes from the diffusion of the upper and lower cartilage endplates. Some studies suggest that higher BMD increases endplate and intradiscal pressure, and the increased static compressive force of the endplate leads to decreased levels of diffusion of nutrients such as glucose into the disc, which promotes IDD [22, 23]. Homminga J et al. believed that with the degeneration of the lumbar intervertebral disc, the water content of the nucleus pulposus decreased, causing fibrosis of the nucleus pulposus, calcification of the cartilage endplates and the formation of surrounding osteophytes. These may reduce the density of the trabecular core while increasing the density of the vertebral cortex [20]. Margulies JY et al. believed that a decrease in lumbar vertebral BMD will lead to a decrease in the number of trabecular bones in the vertebral body and an increase in bone fragility, thereby causing microfractures under the superior endplate of the lumbar spine. It affected the nutrient supply within the lumbar spine and upper intervertebral disc, thereby promoting IDD [24]. There are few theories on the correlation between BMD difference and IDD. However, we believe that multiple theories and mechanisms, including the BMD mentioned above, and IDD correlation hypothesis, the biomechanics and mechanism of pressure load transmission between disc and vertebral body, and the effect of a difference in the BMD loss rate at different levels on IDD need to be integrated to explain our findings.

This study also has some limitations. This study is a small sample study with a large age span. In this study, DXA was used for BMD measurement. The lumbar spine image obtained by it is a two-dimensional plane projection, which cannot effectively distinguish cortical bone and cancellous bone. In addition, bone hyperplasia, IDD and spondylolisthesis will affect the accuracy of BMD.

## Conclusions

For postmenopausal women, an increase in BMD difference is correlated with IDD. For postmenopausal women, osteoporosis is more common in people over 60 years old, and the possibility of modic change in 71-80y is higher than in other age groups. The incidence of severe IDD also increases with aging, especially for the L1/2 and L2/3 discs.

## Data Availability

The data sets cannot be made publicly available, and restrictions apply to the availability of these data. Yihui Zhang should be contacted if someone wants to request the data from this study.

## Abbreviations

LIDD: Lumbar intervertebral disc degeneration
BMD: Bone mineral density
IDD: Intervertebral disc degeneration
T: Thoracic
L: Lumbar
MRI: Magnetic resonance imaging
QA: Quality assurance
CT: Computed tomography
DXA: Dual-energy X-ray absorptiometry
